# Trifluoromethanesulfonamide Induces Male Sterility Through Systemic Metabolic Reprogramming and Anther-Specific Proline Deficiency

**DOI:** 10.3390/ijms27125554

**Published:** 2026-06-19

**Authors:** Yuka Sekiguchi, Yan Gao, Hiromitsu Tabeta, Muneo Sato, Masami Yokota Hirai, Nasrein Mohamed Kamal, Takayoshi Ishii

**Affiliations:** 1 The United Graduate School of Agricultural Sciences, Tottori University, 4-101 Koyama Minami, Tottori 680-8550, Japan; d23a3111z@edu.tottori-u.ac.jp (Y.S.); d23a3003b@edu.tottori-u.ac.jp (Y.G.); 2 Research Fellow of Japan Society for the Promotion of Science DC, 5-3-1, Kojimachi, Chiyoda-Ku, Tokyo 102-0083, Japan; 3 RIKEN Center for Sustainable Resource Science, 1-7-22, Suehiro-cho, Tsurumi-Ku, Yokohama 230-0045, Japan; hiromitsu.tabeta@riken.jp (H.T.); muneo.sato@riken.jp (M.S.); masami.hirai@riken.jp (M.Y.H.); 4 Graduate School of Agricultural Science, Kobe University, 1-1, Rokkodai-cho, Nada-Ku, Kobe 657-8501, Japan; 5 Agricultural Research Corporation (ARC), Wad Medani P.O. Box 126, Sudan; renokamal@tottori-u.ac.jp; 6 International Platform for Dryland Research and Education (IPDRE), Tottori University, 1390, Hamasaka, Tottori 680-0001, Japan; 7 Arid Land Research Center (ALRC), Tottori University, 1390, Hamasaka, Tottori 680-0001, Japan; 8 Chromosome Engineering Research Center, Tottori University, 86 Nishi-Cho, Yonago 683-8503, Tottori, Japan

**Keywords:** chemical hybridization agent, dryland, pollen viability, proline metabolism, reproductive development, amino acid metabolism

## Abstract

Chemical hybridization agents (CHAs) enable efficient, large-scale hybrid seed production, yet their mechanisms remain poorly understood. Understanding how CHAs induce male sterility at the metabolic level is important for both basic pollen biology and crop breeding. Here, we performed integrated metabolomic analyses to investigate the metabolic basis of the action of trifluoromethanesulfonamide (TFMSA) across multiple species and tissues. TFMSA treatment induced systemic metabolic reprogramming across species, prominently affecting amino acid metabolism, central carbon metabolism, and one-carbon metabolism. Although individual metabolite responses varied among species, pathway-level analyses consistently revealed coordinated modulation of carbon–nitrogen metabolic networks. In reproductive tissues, TFMSA induced tissue-specific metabolic changes. In cowpea anthers, proline was the only metabolite significantly altered and was strongly depleted, whereas in floral tissues several amino acids, including phenylalanine and tyrosine, were accumulated. Pathway analysis revealed altered amino acid metabolism, suggesting that systemic metabolic responses accompanied the proline reduction in anthers. These findings indicate that TFMSA induces male sterility through coordinated metabolic reprogramming across tissues and species, leading to depletion of key metabolites required for pollen development. This study provides a metabolic framework for understanding CHA-induced male sterility and highlights TFMSA as a powerful tool for probing metabolic regulation of pollen development.

## 1. Introduction

Emasculation refers to the induction of male sterility in plants to enable crossing between different individuals and the creation of new genetic combinations. Several emasculation methods have been developed, including manual emasculation, cytoplasmic male sterility, hot water treatment, and the use of chemical hybridization agents (CHAs), each with inherent limitations [1,2,3,4,5,6]. Manual emasculation is labor-intensive and requires extensive training, while cytoplasmic male sterility relies on specific nuclear–mitochondrial genome combinations and is therefore restricted to particular species and genotypes. In contrast, CHAs allow simultaneous treatment of large numbers of plants, substantially reducing labor requirements [7]. However, their effectiveness is often species-dependent, with compounds that induce male sterility in one species often failing to produce comparable effects in others [8,9,10].

Various CHAs have been used to induce male sterility in both dicotyledonous and monocotyledonous plants, and their mechanisms frequently involve broad metabolic disruptions [11]. In dicot species, sulfonylurea-type CHAs that act as acetolactate synthase inhibitors are widely used [12]. For example, tribenuron-methyl induces male sterility in *Brassica* species by inhibiting branched-chain amino acid biosynthesis, resulting in depletion of valine, leucine, and isoleucine and subsequent autophagic cell death in anthers [10,13]. Monosulphuron ester sodium similarly disrupts branched-chain amino acid synthesis and interferes with tapetal and microspore development in *Brassica napus*, with proteomic analyses revealing altered expression of proteins involved in energy metabolism and amino acid pathways [9]. In addition, tribenuron-methyl has been reported to suppress flavonoid biosynthesis, disturb protein processing in the endoplasmic reticulum, and cause defective tapetal development [14]. Together, these studies suggest that CHA-induced male sterility in dicots frequently involves disruption of amino acid metabolism, secondary metabolite biosynthesis, and cellular energy homeostasis.

In monocot crops such as wheat, the CHA sintofen has been extensively studied [15,16,17]. Proteomic and physiological analyses indicate that sintofen treatment disrupts carbohydrate metabolism, reduces starch accumulation, alters soluble sugar balance, and induces oxidative stress during anther development [15,16]. Metabolomic analyses further demonstrate perturbations in central carbon metabolism and energy-related pathways in sterile anthers [18]. Although the primary metabolic targets differ between dicotyledonous and monocotyledonous species, these studies collectively suggest that CHA-induced male sterility commonly involves extensive metabolic disturbances during anther and pollen development.

Trifluoromethanesulfonamide (TFMSA) is widely used in chemistry as a catalyst, solvent, acid titrant, and reagent in peptide synthesis. More recently, it has been applied in plant breeding as a CHA capable of inducing male sterility without severely affecting plant growth when used at appropriate dosages [8,19,20]. TFMSA has demonstrated efficacy across multiple plant species, including maize, sorghum, cowpea, tobacco, and *Arabidopsis thaliana* [8,19,20].

In maize, TFMSA-induced male sterility has been linked to reduced proline transport from glumes to developing anthers, which may lead to feedback inhibition of proline biosynthesis [8]. However, Mattioli et al. [21] demonstrated that local proline biosynthesis within developing microspores and mature pollen grains contributes more strongly to pollen proline pools than transport from surrounding sporophytic tissues and is essential for pollen fertility in *A. thaliana*. Consistent with this finding, genetic analyses have shown that single, double, or triple knockout mutants of the proline transporter genes *AtProT1*, *AtProT2*, and *AtProT3* do not show reduced proline accumulation or impaired pollen germination [22]. Thus, inhibition of proline transport alone cannot fully explain TFMSA-induced pollen sterility in *A. thaliana*.

Despite the broad applicability of TFMSA, the metabolic mechanisms underlying TFMSA-induced male sterility across diverse plant species remain poorly understood. Pollen development depends on tightly regulated metabolic processes, including carbon and amino acid metabolism and the maintenance of cellular redox balance [16,21,23]. Analyzing these metabolic changes can offer valuable insights into the mechanisms behind TFMSA-induced male sterility. Here, we present a comparative, systems-level analysis of TFMSA-induced metabolic changes in monocotyledonous and dicotyledonous species, providing insights into how conserved metabolic disruptions and species-specific responses together contribute to the induction of male sterility. In particular, we highlight tissue-specific metabolic responses in reproductive organs, where tightly regulated metabolism is essential for proper pollen development. By integrating metabolomic analyses across multiple species and tissue types, we demonstrate that both systemic and tissue-specific metabolic changes contribute to TFMSA-induced male sterility.

## 2. Results

### 2.1. TFMSA Induces Male Sterility Across Diverse Plant Species Through Spray and Soil Application

To evaluate the applicability of TFMSA across a wide range of plant species, treatments were applied using either spray or soil application methods. Pollen viability was assessed using Alexander staining, seed set rate measurements or flow cytometric analysis. Spray application induced male sterility in species belonging to *Brassicaceae*, *Fabaceae*, *Solanaceae,* and *Poaceae*, although the required dosage varied among species, ranging from 1 to 60 mg per plant (Table S1). Soil application also induced male sterility in species of *Brassicaceae*, *Fabaceae*, and *Solanaceae*, with effective dosages ranging from 0.2 to 30 mg per plant (Table S1).

**Figure 1:**
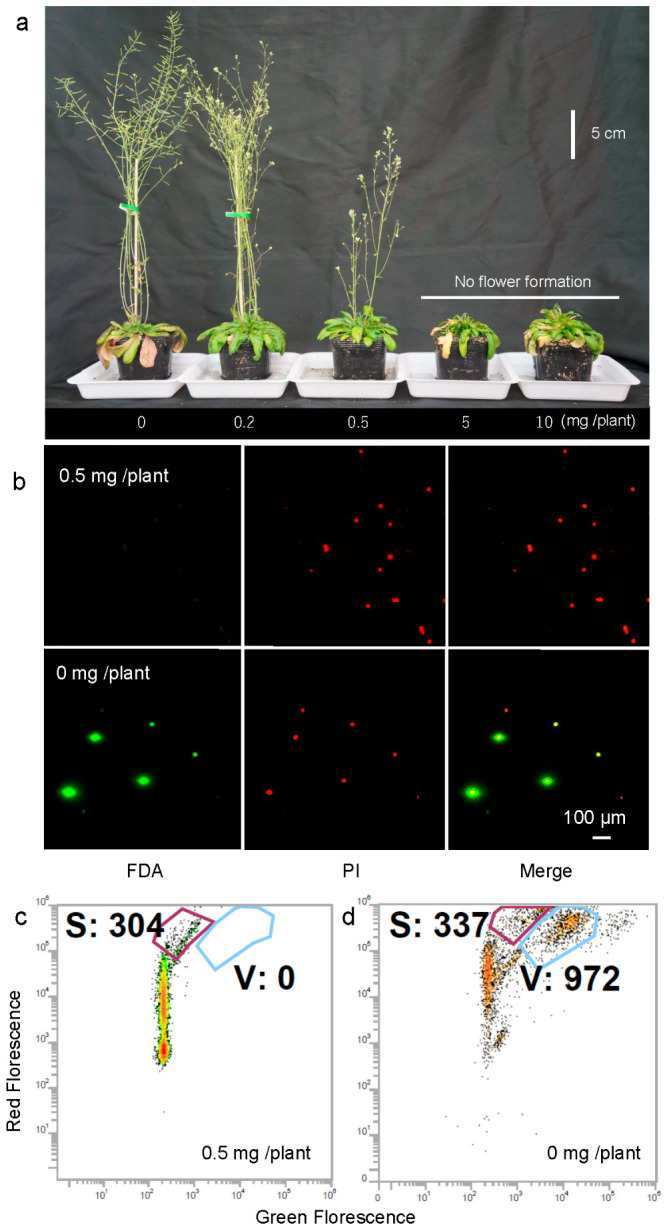
Pollen fertility and plant growth in *A. thaliana* after TFMSA treatment via soil application. (**a**) Plant growth was suppressed in a dose-dependent manner. (**b**) Fluorescence microscopy images of pollen grains from plants treated with 0 or 0.5 mg TFMSA per plant, stained with fluorescein diacetate (FDA) and propidium iodide (PI). Viable pollen grains exhibit green fluorescence (FDA), whereas non-viable pollen grains show red fluorescence (PI). (**c**) Pollen grains from plants treated with 0.5 mg TFMSA per plant used for flow cytometry analysis. A total of 304 pollen grains were sterile (S), and no viable pollen grains (V) were detected. (**d**) Pollen grains from plants treated with 0 mg TFMSA per plant used for flow cytometry analysis. A total of 337 pollen grains were sterile (S), while 972 were viable (V). The X- and Y-axes are displayed on a logarithmic scale.

Detailed pollen analysis was conducted following soil application of TFMSA using fluorescence microscopy and flow cytometry. In diploid *A. thaliana*, soil treatment with 0.5 mg TFMSA resulted in predominantly red fluorescence when stained with propidium iodide (PI), indicating non-viable pollen, and almost all detected pollen grains were classified as non-viable (974/975; 100% sterility) (Figure 1, Table S2). In contrast, control diploid *A. thaliana* plants produced a total of 3450 pollen grains, of which 2563 (74%) were viable and 887 (26%) were non-viable (Table S2).

Overall, TFMSA treatment consistently impaired pollen function across these evolutionary diverse species, although the effective dosage differed among species (Table S1). These observations raised the question of how TFMSA induces male sterility across such diverse taxa. To address this, we selected cowpea, *A. thaliana*, and sorghum as representative species to conduct comprehensive metabolomic analyses to investigate TFMSA-induced metabolic changes across species and tissues. Those three species represent distinct taxonomic groups and exhibited strong and reproducible responses to TFMSA treatment.

### 2.2. TFMSA Treatment Alters Leaf Metabolic Profiles in Cowpea, A. thaliana, and Sorghum

To examine metabolic responses to TFMSA treatment at the leaf level, metabolomic analyses were performed in cowpea, *A. thaliana*, and sorghum. A total of 101 metabolites were detected in cowpea, 105 in *A. thaliana*, and 94 in sorghum, of which 49 were commonly identified in all species (Table S3). After interquartile range–based filtering, 46 metabolites remained and were subjected to principal component analysis and hierarchical clustering.

Principal component analysis clearly separated TFMSA-treated samples from control samples within each species, indicating that TFMSA substantially altered leaf metabolic profiles (Figure S1). Two-way ANOVA using species and treatment as factors revealed both conserved and species-specific metabolic responses to TFMSA (Table S4). Six metabolites showed significant treatment main effects, and 32 metabolites showed significant species × treatment interaction effects (Table S4).

**Figure 2:**
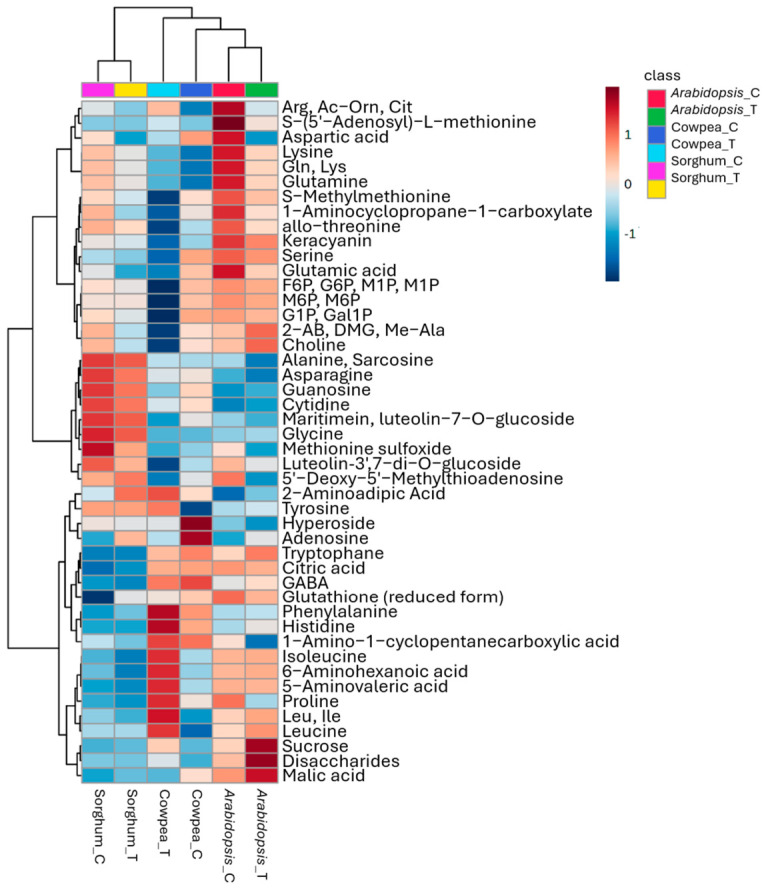
Hierarchical clustering heatmap of metabolite profiles with or without TFMSA treatment. Rows represent metabolites, and columns represent the average of biological replicates (*n* = 3–10). Metabolite abundances were normalized and Z-score scaled across samples. Red indicates higher relative abundance, and blue indicates lower relative abundance. Sample classes are defined as follows: C, control; T, TFMSA-treated.

Hierarchical clustering of metabolite profiles further revealed a clear separation between control and TFMSA-treated samples. Although samples of the same species tended to cluster together, clustering patterns were not strictly species-dependent (Figure 2).

Metabolites significantly altered by TFMSA treatment totaled 53 in cowpea, 37 in *A. thaliana*, and 8 in sorghum (Table S5). Significance was determined using FDR-adjusted *p* values < 0.05 for cowpea and *A. thaliana*, and a raw *p* value threshold of <0.05 for sorghum. In cowpea, four metabolites increased (fold change 2.4–8.9), including phenylalanine, tyrosine, leucine, and proline. In contrast, 49 metabolites significantly decreased (fold change 0.018–0.44), including several amino acids and related metabolites, intermediates of central carbon metabolism such as succinic acid and 2-oxoglutaric acid, nucleotide-related metabolites including uridine-5′-monophosphate and β-nicotinamide mononucleotide, and phenylpropanoid-related metabolites such as ferulate and 4-coumaric acid (Table S5).

In *A. thaliana*, 30 metabolites were significantly increased following TFMSA treatment (fold change 2.1–9.2). These included several amino acids such as phenylalanine, tyrosine, tryptophan, histidine, leucine, and isoleucine, as well as metabolites associated with carbohydrate metabolism (e.g., sucrose, raffinose, and kestose), nucleotide metabolism (cytidine and guanosine), and cofactor metabolism (β-nicotinamide mononucleotide and pantothenate). Several glucosinolates, including 1-methoxyindole-glucosinolate and 4-methoxyindole-glucosinolate, showed significant accumulation. Seven metabolites decreased (fold change 0.22–0.46), including glutamine, lysine, sinapoyl malate, and several glucosinolates (Table S5).

In sorghum, eight metabolites were significantly increased following TFMSA treatment (fold change 1.6–5.2). These included metabolites associated with lysine metabolism, such as 2-aminoadipic acid; central carbon metabolites including 2-oxoglutaric acid; nucleotide-related metabolites such as inosine and 5′-deoxy-5′-methylthioadenosine; and several osmoprotectant or cofactor-related compounds including betaine, trigonelline, and pyridoxal-5′-phosphate. Rosmarinic acid also showed significant accumulation. These results indicate that both the number and identity of TFMSA-responsive metabolites differed markedly between species, while showing similar trends among dicots (Table S5). TFMSA induces both common and specific metabolic responses across the three plant species. Although all species show changes in amino acid, nucleotide, and stress-related pathways, their responses range from significant metabolic depletion in cowpea to compensatory accumulation in *A. thaliana* and minimal adaptive stress responses in sorghum. Metabolites significantly affected by TFMSA were subsequently used to identify associated metabolic pathways.

### 2.3. Pathway Enrichment Analysis of Leaf Metabolites After TFMSA Treatment

Pathway enrichment analysis was conducted using all significantly altered metabolites detected in each species, rather than only metabolites shared across species (Table S6). Several metabolic pathways were significantly enriched following TFMSA treatment detected by raw *p* < 0.05 threshold, and significance was further evaluated using FDR-adjusted *p*-values.

In cowpea leaves, 11 metabolic pathways were affected, including multiple amino acid metabolism pathways such as glycine–serine–threonine metabolism; alanine–aspartate–glutamate metabolism, cysteine–methionine metabolism, cyanoamino acid metabolism, and arginine biosynthesis. In addition, several pathways related to central carbon and carbohydrate metabolism were enriched, including glyoxylate, dicarboxylate, and butanoate metabolism. Enrichment was also observed in nicotinate and nicotinamide metabolism, phenylpropanoid biosynthesis, and glycerolipid metabolism, and the one-carbon pool by folate pathway (Table S6).

In *A. thaliana*, six pathways were significantly enriched. These included amino acid biosynthesis pathways (alanine, aspartate, phenylalanine, tyrosine, and tryptophan) as well as lysine degradation. Enrichment was also observed in nicotinate and nicotinamide metabolism and the one-carbon pool by folate pathway (Table S6).

In contrast, sorghum showed a weaker pathway-level response. Only one pathway (vitamin B6 metabolism) reached statistical significance (Table S6).

To investigate tissue-specific metabolic responses to TFMSA, we further analyzed cowpea flower buds. Cowpea reproductive tissues were collected from plants grown in the field, while leaf metabolomic analyses in the comparative species experiments were performed under controlled conditions. Consequently, environmental factors should be taken into account when directly comparing the metabolic responses in leaf and reproductive tissue datasets. Cowpea was selected because of its consistent response to TFMSA and the availability of clearly defined flower developmental stages, allowing the collection of sufficient amounts of anther tissue for metabolomic analysis with high reproducibility, which facilitates investigation of anther and pollen development.

### 2.4. Metabolomic Changes in Anthers and Flowers Following TFMSA Treatment in Cowpea

We analyzed 74 metabolites in reproductive tissues, of which 43 were detected in both flowers and anthers. TFMSA treatment induced significant tissue-specific metabolic changes in cowpea.

**Figure 3:**
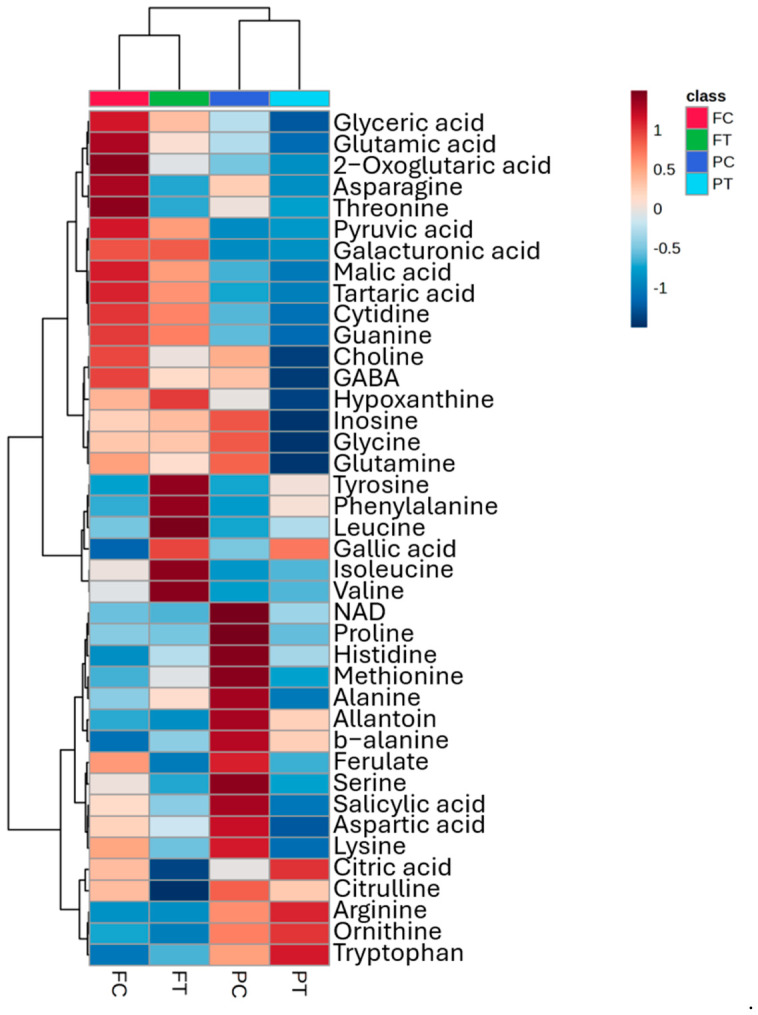
Hierarchical clustering heatmap of metabolite profiles in anthers and flowers of cowpea with or without TFMSA treatment. Rows represent metabolites, and columns represent the average of individual biological replicates. Metabolite abundances were normalized and Z-score scaled across samples. Red indicates higher relative abundance, and blue indicates lower relative abundance. Sample classes are defined as follows: FC, control flowers; FT, TFMSA-treated flowers; PC, control anthers; PT, TFMSA-treated anthers.

Principal component analysis clearly separated TFMSA-treated samples from control samples in both anthers and flowers, indicating distinct metabolic alterations within each tissue (Figure S2). Hierarchical clustering of metabolite profiles further revealed strong separation between anther and flower tissues regardless of treatment, reflecting inherent metabolic differences between these tissues (Figure 3).

Two-way ANOVA using tissue type (anther vs. flower) and treatment as factors revealed significant treatment effects for several metabolites, including phenylalanine, tyrosine, tryptophan, leucine, isoleucine, valine, histidine, and galacturonic acid (Table S7). Significant tissue × treatment interactions were detected for proline, methionine, malic acid, and citric acid. Among these, proline showed a particularly strong interaction effect, being significantly depleted in anthers but not in flowers following TFMSA treatment.

Several metabolites, including glutamic acid, glutamine, lysine, arginine, glycine, serine, and nicotinamide adenine dinucleotide, differed between tissues but did not respond significantly to TFMSA treatment (Table S7).

Fold-change analysis revealed clear tissue-dependent responses to TFMSA treatment (Table S8). Significance was determined using FDR-adjusted *p* values < 0.05. In anthers, proline showed a pronounced reduction (fold change = 0.10; log_2_ fold change = −3.3). In contrast, four metabolites significantly changed in flowers (fold changes of 2.0–7.8), where several amino acids, including the aromatic amino acids such as phenylalanine and tyrosine and the branched-chain amino acid leucine, showed strong accumulation. Notably, the severe depletion of proline observed in anthers was not detected in flowers (Table S8). These results indicate a distinct tissue-specific metabolic response characterized by proline depletion in anthers and amino acid accumulation in flowers.

### 2.5. Pathway Enrichment Analysis in Cowpea Reproductive Tissues

Pathway enrichment analysis of significantly altered metabolites revealed tissue-specific metabolic responses in reproductive tissues (Table S9). Significance was detected by raw *p* < 0.05 threshold, and was further evaluated using FDR-adjusted *p*-values.

In flowers, enriched pathways were primarily related to amino acid metabolism, including phenylalanine, tyrosine, and tryptophan biosynthesis as well as arginine and proline metabolism (Table S9). These pathways correspond to the accumulation of amino acids such as phenylalanine, tyrosine, and leucine in floral tissues. In contrast, pathway analysis could not be performed in anthers because proline was the only metabolite that showed a significant difference (Table S9).

**Figure 4:**
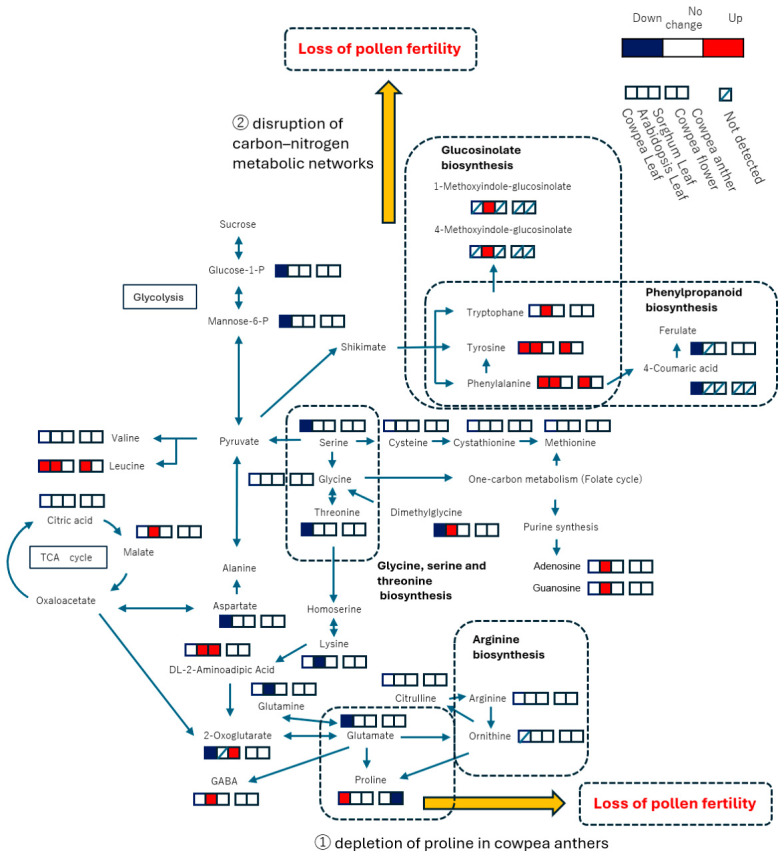
TFMSA-induced metabolic changes in reproductive and leaf tissues. Schematic representation of central carbon, amino acid, and one-carbon metabolic pathways affected by TFMSA treatment. Metabolites showing significant changes are indicated by colored boxes. Color scale represents log_2_ fold change values relative to control (blue, decreased Log_2_ FC < 0; red, increased Log_2_ FC > 0; no color, not detected as significant change).

These tissue-specific responses were integrated with the systemic metabolic changes observed in leaves. While leaves exhibited broad metabolic changes involving amino acid metabolism, central carbon metabolism, and related pathways, reproductive tissues showed more targeted alterations. The overall metabolic response to TFMSA across species and tissues is summarized in Figure 4.

Figure 4 supports a model in which TFMSA induces proline depletion in anthers, triggering systemic metabolic perturbations across interconnected carbon–nitrogen networks. The pathway-level responses collectively suggest that TFMSA treatment induces coordinated metabolic reprogramming characterized by enhanced amino acid accumulation and reduced flux through central carbon metabolism. These changes include widespread alterations in central carbon metabolism, such as glycolysis and the tricarboxylic acid cycle, which are closely linked to amino acid biosynthesis pathways. Perturbations were also observed in multiple amino acid metabolic pathways, including glycine–serine–threonine metabolism, arginine biosynthesis, and aromatic amino acid biosynthesis via the shikimate pathway.

Together, these results indicate that TFMSA induces coordinated metabolic perturbations at the whole-plant level while imposing a strong metabolic limitation in anthers, especially through the depletion of proline.

## 3. Discussion

### 3.1. Metabolic Changes Underlying TFMSA-Induced Male Sterility

In this study, we investigated the metabolic basis of TFMSA-induced male sterility in cowpea, *A. thaliana*, and sorghum using leaf and flower tissues. Unlike earlier models that primarily attributed TFMSA-induced sterility to inhibition of proline transport [8], our results demonstrate that TFMSA induces systemic metabolic changes across multiple interconnected pathways in different species and tissues.

The classical model proposed in maize suggests that TFMSA inhibits proline transport from glumes to developing anthers, leading to feedback inhibition of proline biosynthesis. However, subsequent studies indicate that local proline biosynthesis within developing microspores plays a dominant role in pollen proline accumulation [21], and that disruption of proline transport alone does not necessarily impair pollen fertility in *Arabidopsis* [22]. These findings suggest that the mechanism of TFMSA-induced male sterility is likely more complex than previously proposed.

Our metabolomic analyses revealed that TFMSA treatment alters several key metabolic pathways, including amino acid metabolism, central carbon metabolism, and one-carbon metabolism. At the leaf metabolite level, TFMSA induced significant accumulation of amino acids, particularly aromatic amino acids. In contrast, many central carbon intermediates, such as organic acids and sugar phosphates, were reduced, indicating a shift in carbon allocation from primary metabolism to amino acid biosynthesis.

Furthermore, metabolites associated with one-carbon metabolism and nucleotide biosynthesis were consistently elevated, indicating enhanced flux through folate-mediated C1 pathways [24]. Although specific metabolite responses varied among species, pathway-level analyses consistently showed coordinated shifts in carbon–nitrogen metabolic networks [25,26].

Species-specific differences in pathway enrichment likely reflect variations in metabolic sensitivity and buffering capacity. In cowpea, the strong involvement of central carbon and amino acid metabolism suggests pronounced metabolic changes that may increase the risk of metabolic imbalance in reproductive tissues. These pathways are critical for energy supply and biosynthetic processes during anther and pollen development [23,27,28]. The enrichment of one-carbon metabolism may reflect activation of compensatory pathways that support nucleotide biosynthesis and cellular proliferation [24]. In contrast, the limited response observed in sorghum, restricted mainly to vitamin B6 metabolism, may reflect a more constrained adjustment related to redox regulation and cofactor-mediated stress responses [29]. Overall, these findings indicate that TFMSA induces metabolic alterations that vary among species but converge in disrupting pollen development. Because male reproductive development requires tightly coordinated carbon supply, amino acid metabolism, and redox regulation, these systemic metabolic changes are likely to have pronounced tissue-specific effects in reproductive organs [30,31]. Since reproductive tissues were gathered from cowpea plants grown in the field, environmental influences cannot be entirely ruled out. Nevertheless, comparable TFMSA-induced metabolic patterns were observed in preliminary experiments carried out under controlled conditions, confirming the reproducibility of the main responses documented in this study.

Male sterility induced by TFMSA likely arises from a combination of conserved metabolic disruptions and species-specific responses, with severity determined by the extent to which these changes interfere with the metabolic demands of the anther. Although core metabolic processes involved in pollen development are broadly conserved, differences in TFMSA sensitivity indicate that species-specific metabolic buffering may influence the outcome [32]. TFMSA disrupts key metabolites, such as proline and carbohydrates [8], and since sugar and lipid metabolism are essential for pollen viability [23], these metabolic disruptions likely have broad consequences for plant reproductive development.

### 3.2. Tissue-Specific Metabolic Limitation in Reproductive Organs

A key finding of this study is the tissue-specific nature of TFMSA-induced metabolic disruption. Whereas leaves and flowers showed accumulation of multiple amino acids, anthers showed a pronounced and specific depletion of proline in cowpea. Two-way ANOVA revealed a significant tissue × treatment interaction for proline, indicating that TFMSA-induced metabolic responses differ among reproductive tissues. Notably, proline levels strongly declined in cowpea anthers, while remaining relatively stable in flowers and leaves.

This tissue-specific depletion is particularly important because proline plays essential roles in pollen development, including osmotic regulation, stress protection, and energy metabolism [21]. However, reduction in proline in male reproductive organs has been observed in maize and cowpea [8]. Further reproductive tissue analyses in additional species will be necessary to determine whether this represents a conserved mechanism underlying TFMSA-induced male sterility across diverse taxa. Nevertheless, the targeted reduction in proline in anthers suggests that TFMSA induces a localized metabolic restriction in tissues with high energy demand, thereby inhibiting pollen development and leading to male sterility. The mechanism underlying the selective depletion of proline in cowpea anthers remains unclear. However, TFMSA may influence proline metabolism or the distribution of proline between vegetative and reproductive tissues, as we observed a strong depletion of proline in excised anthers of TFMSA-treated cowpea, whereas proline accumulated in leaf tissues.

### 3.3. Comparison with the Metabolic Mechanisms of Other CHAs

Previous studies have shown that CHAs such as acetolactate synthase inhibitors and sintofen induce male sterility in both dicot and monocot species by disrupting amino acid metabolism, carbohydrate metabolism, and cellular energy homeostasis [9,10,15,16]. Consistent with these studies, our results indicate that TFMSA also affects multiple metabolic pathways associated with primary metabolism.

In our study, however, a critical tissue-specific disruption was observed in cowpea anthers, where proline was markedly depleted. Proline plays key roles in pollen development, including osmotic regulation, maintenance of redox balance, and serves as an energy and carbon source during microspore maturation [21,33]. Disruption of proline metabolism has also been linked with defects in developmental processes because of its central role in cellular redox homeostasis and metabolic flux regulation [34].

The specific depletion of proline in anthers likely imposes a localized metabolic constraint that cannot be compensated by surrounding floral tissues. This limitation may contribute to the observed shift in carbon allocation from primary metabolism toward amino acid pools [35]. Moreover, the localized nature of this metabolic disruption may explain why TFMSA exerts relatively mild effects on overall plant growth, allowing its use as a breeding tool across a diverse range of species compared with other CHAs. Together with the systemic accumulation of amino acids and reduction in central carbon metabolites, these findings suggest that TFMSA induces both global metabolic reprogramming and targeted disruption within reproductive tissues.

Collectively, our results support the general concept that CHA-induced sterility arises from coordinated metabolic disruption while also highlighting a distinctive tissue-specific mechanism for TFMSA involving proline depletion in anthers.

### 3.4. Revised Model of TFMSA-Induced Male Sterility

Based on our present findings, we propose an updated mechanistic model for TFMSA-induced male sterility. TFMSA induces male sterility primarily through local depletion of proline within anthers, creating a critical metabolic limitation in reproductive tissues. Because pollen development depends on tightly regulated metabolic processes, this localized disruption impairs microspore development and ultimately results in pollen sterility.

This anther-specific metabolic constraint is accompanied by systemic metabolic changes throughout the plant, affecting interconnected carbon–nitrogen metabolic pathways. In leaves and floral tissues, these changes are reflected in widespread changes in amino acid metabolism, including accumulation of several amino acids and reductions in central carbon metabolites, suggesting a redistribution of carbon and nitrogen resources [26].

TFMSA-induced male sterility should therefore be viewed not simply as the consequence of inhibited proline transport but rather as the outcome of a localized metabolic constraint in anthers coupled with broader metabolic reorganization across the whole plant. The present findings provide a framework for understanding how TFMSA induces male sterility through coordinated metabolic disruption and tissue-specific proline depletion in anthers. Time-course metabolomic and transcriptomic analyses will be necessary to clarify the dynamic progression of TFMSA-induced metabolic reprogramming in plant in the future. Improved understanding with multi omics data for the TFMSA induced male sterility in plants may contribute not only to the development of more effective and broadly applicable chemical hybridization agents, but also to a deeper understanding of the pollen development and fertility in plants.

## 4. Material and Methods

### 4.1. Plant Materials and Growth Conditions

Diploid *Arabidopsis thaliana* L. (Col-0, accession 140931, 2*n* = 2*x* = 10), tetraploid *A. thaliana* L. (Col-0, accession N3151, 2*n* = 4*x* = 20), and *A. suecica* (Fr.) Norrl. (WU9510, 2*n* = 4*x* = 26) were germinated and grown in pots (7.5 cm diameter × 6.5 cm height) filled with a planting soil mixture (Takii-Tanemaki-Baido, Cainz Co., Saitama, Japan) in a growth chamber under a 12 h/12 h (day/night) photoperiod at 26 °C/18 °C (day/night). Five to seven days after germination, plants were transferred to a vernalization chamber under a 9 h/15 h (day/night) photoperiod at 4 °C for one month. After vernalization, plants were transplanted individually and grown in a plant growth room under a 9 h/15 h (day/night) photoperiod at 26 °C/18 °C (day/night). After flower bud formation, plants were transferred to a flowering room maintained under a 12 h/12 h (day/night) photoperiod at 26 °C/18 °C (day/night).

Soybean (*Glycine max* L. cv. Umai-Chamame, 2*n* = 2*x* = 40, Tohoku Seed Co., Ltd., Utsunomiya, Japan), pea (*Pisum sativum* L. cv. Takasasaendo, 2*n* = 2*x* = 14, Asahi Noen Seed Co., Ltd., Inazawa, Japan), petunia (*Petunia hybrida* L. cv. Pastel Color Mix, 2*n* = 2*x* = 14, Sakata Seed Corporation, Yokohama, Japan), and tobacco (*Nicotiana tabacum* L., 2*n* = 4*x* = 38) were sown in 72-cell trays (Tokaikasei Co., Ltd., Gifu, Japan) filled with granular culture soil (Nippi-Engei-Baido; Nihon Hiryo Co., Ltd., Tokyo, Japan) and grown in a growth chamber under a 12 h/12 h (day/night) photoperiod at 26 °C with 40%/60% (day/night) humidity.

Cowpea (*Vigna unguiculata* L., accession IT97K-499-35, 2*n* = 2*x* = 22), sorghum (*Sorghum bicolor* (L.) Moench, accession AG8, 2*n* = 2*x* = 20), and wheat (*Triticum aestivum* L. cv. Chinese Spring, 2*n* = 6*x* = 42) seeds were directly sown in pots (9 cm diameter × 20 cm height) filled with Nippi-Engei-Baido.

Tomato (*Solanum lycopersicum* L.cv. Niagara Sweet, 2*n* = 2*x* = 24, Sanyo Seed Co., Ltd., Japan), eggplant (*Solanum melongena* L. cv. Suzunari Long Eggplant, 2*n* = 2*x* = 24, ITANSE Co., Ltd., Osaka, Japan), and pepper (*Capsicum annuum* L. cv. Suzunari Green Pepper, 2*n* = 2*x* = 24, ITANSE Co., Ltd., Osaka, Japan) seedlings were purchased and grown in pots filled with Nippi-Engei-Baido in a greenhouse under a 16 h/8 h (day/night) photoperiod at 26 °C/18 °C (day/night).

Cowpea (*Vigna unguiculata* L. cv. Sasaque, 2*n* = 2*x* = 22) was directly sown in the field and grown under field conditions from August to September 2025 at the Arid Land Research Center of Tottori University, Japan.

### 4.2. TFMSA Treatment

TFMSA (Tokyo Chemical Industry Co. Ltd., Tokyo, Japan) was dissolved in tap water containing Approach BI (0.1% *v*/*v*; Maruwa Biochemical Co. Ltd., Tokyo, Japan) as a spreading agent at concentrations ranging from 0 to 3000 mg L^−1^.

To evaluate the applicability of TFMSA across evolutionarily diverse plant species, the following species were treated by spray or soil application: diploid and tetraploid *A. thaliana*, *A. suecica*, cowpea, soybean, pea, petunia, tobacco, tomato, eggplant, pepper, and wheat. Treatments were performed during the vegetative stage before flower bud formation.

TFMSA solution was applied via soil application to diploid *A. thaliana,* cowpea, pea, soybean, tomato, eggplant, and pepper. Spray application was used for diploid and tetraploid *A. thaliana*, *A. suecica*, cowpea, petunia, tobacco and wheat.

For metabolomic analysis in sorghum, 20 mg of TFMSA was dissolved in 10 mL of water and applied to the soil surface 25 days after sowing. In cowpea, 30 mL of a 1000 mg L^−1^ TFMSA solution was sprayed onto the plants. In *A. thaliana*, 0.5 mg of TFMSA was dissolved in 10 mL of water and applied via soil application. We selected a single dose that consistently caused significant male sterility while preserving sufficient plant viability for metabolomic analysis.

### 4.3. Pollen Viability Analysis

Anthers were collected from flower buds one day before anthesis and fixed in ethanol:glacial acetic acid (3:1, *v*/*v*) for 2 to 3 days at room temperature, then stored at 4 °C until use. Anthers were stained with Alexander staining solution following Sekiguchi et al. [19], based on the methods of [36,37].

Flow cytometry was used to assess pollen viability. Pollen germination medium was prepared according to Brewbaker and Kwack [38]. Fresh inflorescences (~10 flowers per sample) were placed in 2 mL tubes containing 2000 µL medium and vortexed for 3 s to release pollen grains (modified from Rutley and Miller [39]). The flowers were then removed from the tube.

Fluorescein diacetate (FDA) (FUJIFILM Wako Pure Chemical Corporation, Osaka, Japan) was dissolved in 250 μL acetone and stored at −20 °C until use. Samples were stained with 0.25 µL FDA and 10 μL propidium iodide (PI) (FUJIFILM Wako Pure Chemical Corporation, Osaka, Japan). The pollen suspension was filtered through a 50 µm CellTrics filter (Sysmex Partec GmbH, Görlitz, Germany) to remove debris and incubated for 10 min at room temperature before analysis using a flow cytometer (CyFlow Cube 6, Sysmex Partec GmbH).

Pollen grains showing high green fluorescence (FDA) and low red fluorescence (PI) were classified as viable, whereas those with the opposite pattern were classified as non-viable. At least 300 pollen grains were analyzed per sample.

### 4.4. Metabolomic Analysis in A. thaliana, Cowpea, and Sorghum Following TFMSA Treatment

Leaf tissues of cowpea, *A. thaliana*, and sorghum were sampled for comprehensive metabolomic analysis to evaluate the effects of TFMSA treatment. All samples were collected 14 days after TFMSA treatment.

For cowpea, the youngest fully expanded terminal leaflets from two individual plants were bulked for each control and treated sample, with four biological replicates. For *A. thaliana*, leaves from three individual plants were bulked per sample for each treatment, with 10 biological replicates. For sorghum, the youngest fully expanded leaves from individual plants were sampled without bulking, with three biological replicates.

For cowpea, flower buds were collected from field-grown plants. Three flowers were collected per sample, with four biological replicates. Samples were separated into anthers and floral tissues and pooled to generate one sample.

Collected samples were freeze-dried for 72 h using a freeze dryer (VD-550R, TAITEC Corporation, Tokyo, Japan). Four milligrams of each powdered sample was extracted using either 0.1% (*v*/*v*) formic acid in 80% (*v*/*v*) methanol or 50% methanol without formic acid, with lidocaine and 10-camphorsulfonic acid added as internal standards. Extraction was performed as described previously [40].

Extracted samples were analyzed using a liquid chromatography-tandem mass spectrometry system (Leaves: Nexera MP with LCMS-8050, Shimadzu Corporation, Kyoto, Japan; flowers: Agilent 6420, Santa Clara, CA, USA). Chromatograms were manually inspected using analysis software (MRMPROBS 3.62), and peak identities were confirmed based on retention times and MRM transitions reported previously [41].

Peak areas were integrated and normalized using internal standards and sample mass, and normalized values were used for subsequent analyses. Peak detection quality was evaluated across all samples, and only metabolites reliably detected with consistent retention times and signal intensities were retained for further analysis.

### 4.5. Data Analysis and Statistics

Detected metabolites were analyzed using MetaboAnalyst 6.0 (https://www.metaboanalyst.ca/ accessed on 3 February 2026) for normalization, multivariate analysis, fold-change analysis and pathway enrichment. To ensure comparability among species, two-way ANOVA was performed in SPSS Statistics version 29.0.2.0 (IBM, Tokyo, Japan) using only metabolites commonly detected across species.

For metabolomic data preprocessing, data were first normalized using either sum or median normalization to reduce systematic variation, followed by log transformation to stabilize variance and approximate a normal distribution. Finally, data were subjected to either auto scaling (mean-centering and standardization) or Pareto scaling (mean-centered and divided by the square root of the standard deviation), to improve data distribution and comparability among metabolites prior to statistical analysis [42]. Volcano plots were used to identify significant changes in metabolites based on fold change and Student’s *t*-test. Principal component analysis (PCA) was performed to assess overall metabolic variation and sample grouping, while hierarchical clustering analysis was conducted using Z-score normalized metabolite abundances to visualize patterns of metabolite accumulation across samples. Pathway enrichment analysis was conducted using the pathway analysis module in MetaboAnalyst 6.0 based on the Kyoto Encyclopedia of Genes and Genomes (KEGG) database (https://www.kegg.jp/ accessed on 3 February 2026). Statistical significance was initially evaluated using raw *p*-values and subsequently adjusted using the Benjamini–Hochberg false discovery rate (FDR) method to control for multiple testing. Significantly altered metabolic pathways were selected using a *p* < 0.05 threshold (*t*-test, control vs. treatment in each species, unadjusted *p*-value), and significance was further evaluated using FDR-adjusted *p*-values (FDR < 0.05).

In pathway enrichment analyses, pathways based on raw *p*-values were treated as exploratory metabolic responses, whereas those significant after FDR correction indicated stronger, more robust perturbations due to TFMSA treatment. Since TFMSA treatment induced relatively modest metabolic changes in sorghum compared with cowpea and *A. thaliana*, metabolic enrichment based on raw *p*-values was additionally examined to avoid overlooking potentially relevant biological responses, and these results were reported as exploratory evidence.

## Data Availability

All data supporting the conclusions of this study are included in this article. All data are available upon request from the corresponding author.

## Supplementary Materials

The following supporting information can be downloaded at: https://www.mdpi.com/article/10.3390/ijms27125554/s1.
