# Supplementary material for: Trifluoromethanesulfonamide Induces Male Sterility Through Systemic Metabolic Reprogramming and Anther-Specific Proline Deficiency
Source: Int J Mol Sci. 2026 Jun 19;27(12):5554. doi: 10.3390/ijms27125554 (PMC13300217; doi:10.3390/ijms27125554)
Supplement: Supplementary file 1 [file ijms-27-05554-s001.zip › Supplymental Figures.pdf]

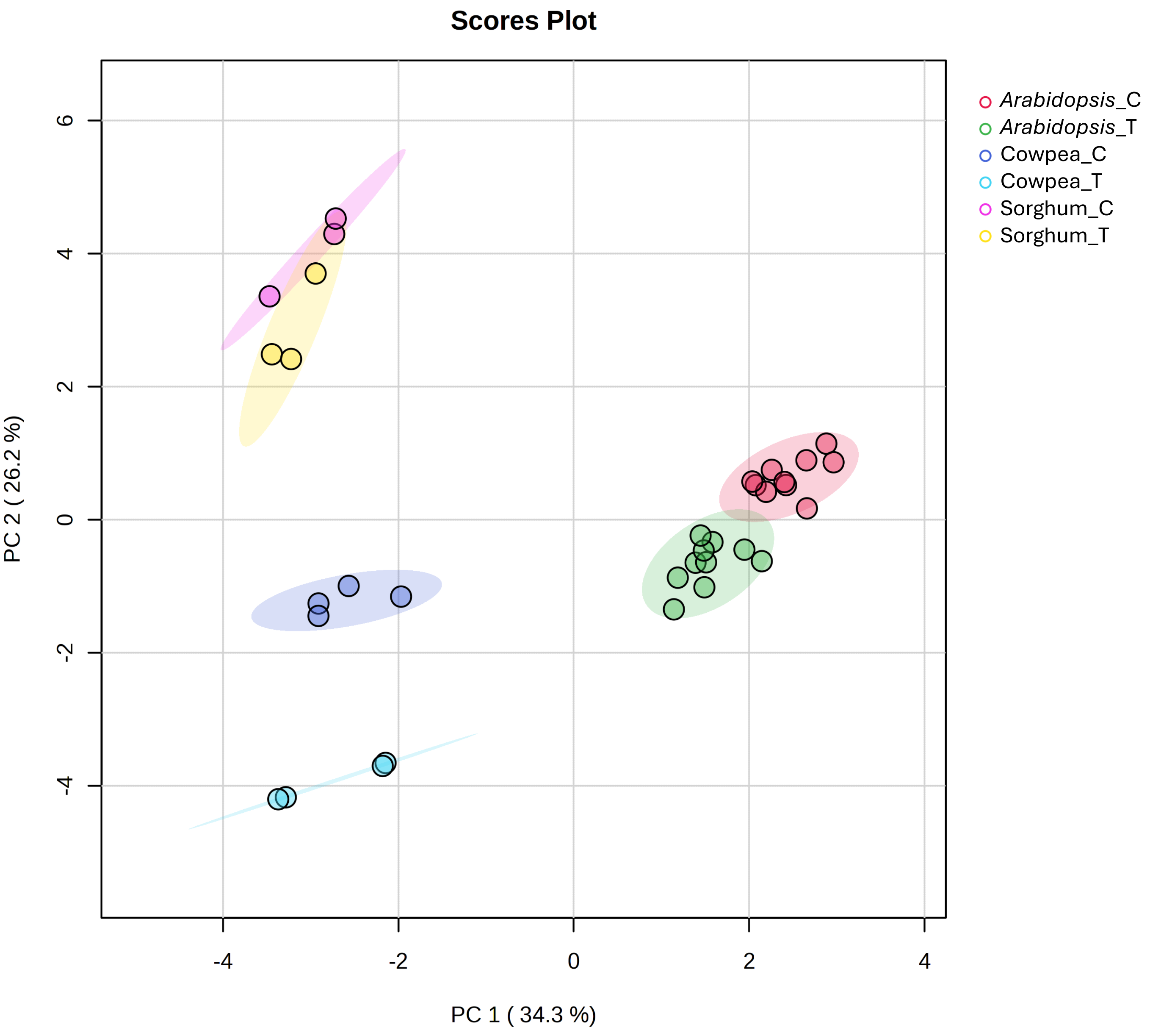

Figure S1 Principal component analysis (PCA) of metabolites between the Control and TFMSA treated leaves of *A.thaliana*, cowpea and sorghum. Data set of 46 commonly identified metabolites are included in the analysis.

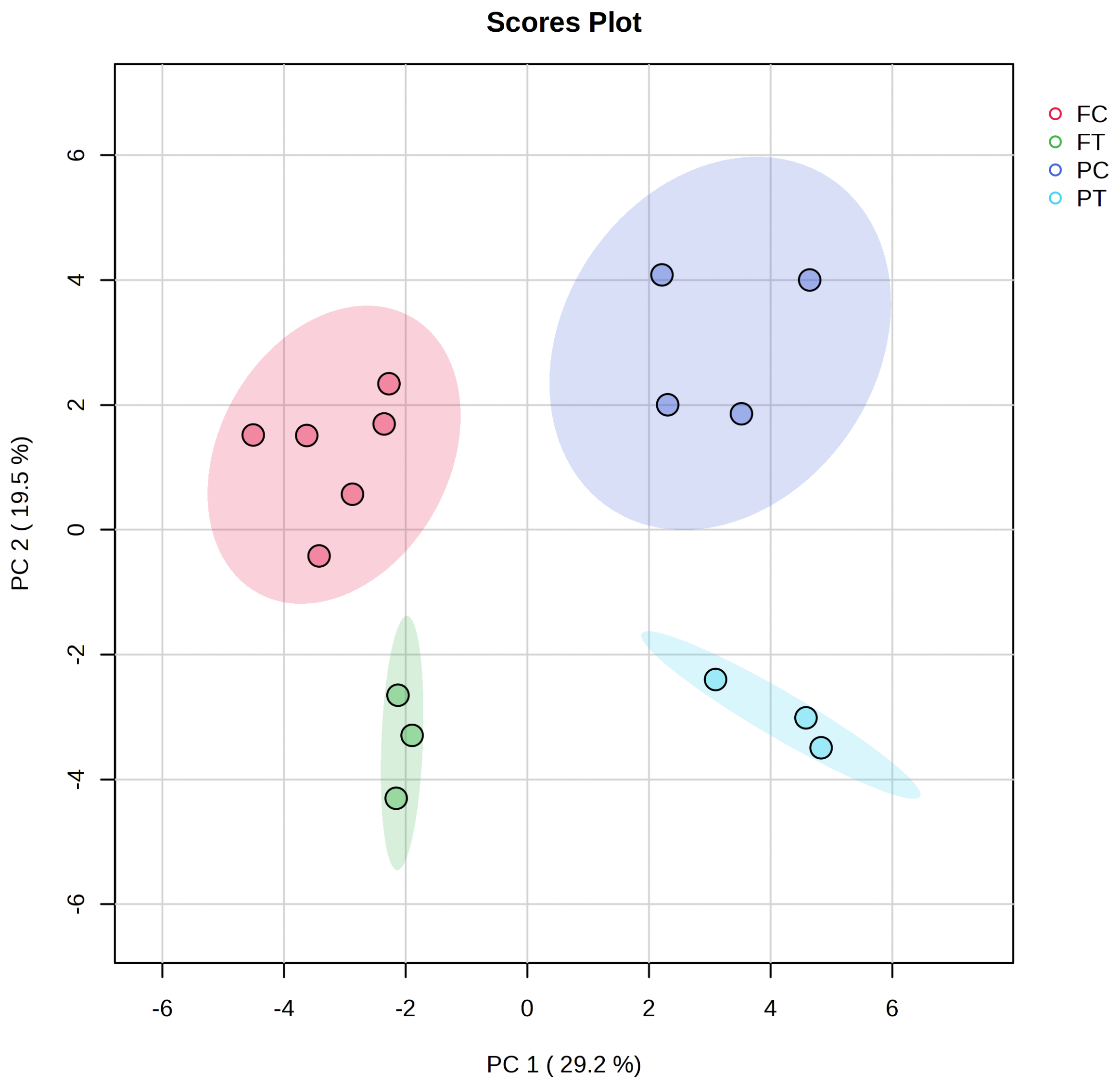

Figure S2 Principal component analysis (PCA) of metabolites between the Control Pollen (PC), TFMSA treated Pollen (PT), Control flower (FC), TFMSA treated flower (FT) of cowpea. One replication consists of flower buds of three plants.
